# Mindfulness as a mediator and moderator between interpersonal alienation and smartphone addiction among Taiwanese university students

**DOI:** 10.1186/s40359-026-04445-1

**Published:** 2026-03-30

**Authors:** Shu-Hsuan Chang, Pi-Kuang Tseng, Jie-Ting Wu, Kai-Jie Chen, Yao-Chung Cheng

**Affiliations:** 1 Department of Electrical and Mechanical Technology, National Changhua University of Education, No.2, Shi-Da Road, Changhua City, Taiwan; 2 Department of Industrial Education, National Taiwan Normal University, No. 162, Sec. 1, He-Ping E. Rd, Taipei, 106209 Taiwan; 3 National Pei-men Senior Agricultural and Industrial Vocational School, No. 117, Liu’an, Jiali Dist., Tainan City, 722012 Taiwan; 4 Graduate Institute of Technology Management, National Chung Hsing University, No.145, Xingda Rd., South Dist., Taichung City, 40227 Taiwan; 5 Center for Teacher Education, National Changhua University of Education, No.1, Jin-De Road, Changhua City, Taiwan; 6 Department of Computer Science and Information Engineering, National Changhua University of Education, Changhua City, Taiwan

**Keywords:** Interpersonal alienation, Mindfulness, Smartphone addiction, Big Five personality traits

## Abstract

**Background:**

Smartphone addiction (SA) is prevalent among university students and is linked to psychosocial and academic risks. Interpersonal alienation (IA) may increase SA via compensatory coping, whereas mindfulness (MI) may serve as a protective self-regulation resource. This study examined the correlations among the Big Five traits, IA, MI, and SA. It tested MI as both a mediator and a moderator in the association between IA and SA.

**Methods:**

A cross-sectional web survey was conducted from May 10–24, 2022, using convenience sampling among Taiwanese university students. The final sample was *N* = 586 from 67 universities, 63.7% female. Measures included BFI-15, IAS, MAAS, and SAS-SV. Confirmatory factor analysis was run in AMOS 25. SPSS 24 supported descriptive statistics, gender comparisons, and Pearson’s r. Mediation was tested using Hayes PROCESS Model 4, and moderation using Model 1.

**Results:**

IA was moderately and positively associated with SA, whereas MI was moderately and negatively associated with both IA and SA. Extraversion and conscientiousness were positively related to MI and negatively related to IA, whereas conscientiousness was also weakly negatively related to SA. Neuroticism was negatively related to MI and positively related to IA and SA; agreeableness showed only small associations, and openness was not significantly related to IA, MI, or SA. Males reported higher IA than females, whereas MI and SA showed no significant gender differences. MI partially mediated the IA–SA association and showed a context-dependent moderation, such that the protective advantage of higher MI diminished as IA increased.

**Conclusions:**

Results supported a compensatory use account in which interpersonal alienation was positively associated with smartphone addiction, whereas mindfulness was negatively associated with both. Mindfulness partially mediated this link and showed a context-dependent moderation, with its protective advantage diminishing as interpersonal alienation increased. Higher education may benefit from scalable mindfulness-based supports combined with efforts to reduce relational disconnection.

## Introduction

Smartphones are integral to daily life and a dominant channel for social connection and communication, but their ubiquity has intensified concern about smartphone addiction as a contemporary social phenomenon [1]. This concern is acute among students, with prevalence estimates ranging from 10% to 44%, underscoring the need to clarify antecedents and feasible intervention points [2]. Evidence associates smartphone addiction with poorer cognitive performance [3], disrupted sleep [4], and heightened anxiety [5] and further links it to academic procrastination [6, 7], lower academic achievement [8], and impaired classroom communication [9]. Interpersonal contexts appear consequential: peer pressure predicts both anxiety and smartphone addiction [10], whereas mindfulness shows a negative association with problematic smartphone use [11]. Together, these findings suggest that smartphone addiction may erode interpersonal functioning and exacerbate social difficulties [12], warranting sustained attention in public discourse and academic inquiry [13].

Recent studies have further clarified the link between smartphone addiction and interpersonal difficulties. For example, Zhao et al. showed that mobile phone addiction often leads to problems in relationships with others, with both loneliness and social anxiety serving as important mediating factors [14]. Their findings indicate that young adults with higher levels of smartphone addiction tend to report more interpersonal problems, not only due to increased loneliness but also because of greater social anxiety [14]. Similarly, Wang et al. identified a positive association between loneliness and smartphone dependence among students [15]. They noted that loneliness can directly contribute to addiction while also reinforcing dependency through processes such as mobile phone anthropomorphism [15]. The quality of interpersonal relationships appears to play a protective role, reducing feelings of loneliness, mobile phone anthropomorphism, and risk of addiction [15]. When loneliness rises, students may turn to their phones for emotional comfort, leading to greater dependence and more unrealistic interactions [15]. Thus, both loneliness and social anxiety not only heighten the risk of interpersonal difficulties but also drive the development of smartphone addiction [14, 15].

The COVID-19 pandemic has amplified these issues. Many people rely on their smartphones for a range of activities to cope with stress, including taking online classes, using social media, shopping online, and gaming. However, extended use during the pandemic has been linked to a higher risk of addiction [16–19]. At the onset of the pandemic, limited access to vaccines and widespread preventive measures such as mask-wearing and social distancing heightened feelings of alienation [20, 21], particularly among students [22]. Such effects may last even after the pandemic.

According to Erikson’s psychosocial development theory, difficulties forming relationships during early adulthood (ages 19–30) can lead to social disconnection and loneliness [23]. Effective communication is closely linked to happiness among university students [24]. Other studies have shown that mindfulness can reduce negative emotions during stress, lower smartphone addiction, and support better relationships [25–27]. In the present study, personality traits are included not as isolated causal drivers but as dispositional background factors that may help explain why some students are more vulnerable than others to interpersonal strain, lower mindfulness, and problematic smartphone use. Recent work further suggests that the link between social relationships and internet-related addiction can be understood through both hedonic and eudaimonic well-being pathways, indicating that relational functioning is embedded in a broader psychosocial system rather than a single risk pathway [28]. The relationship between interpersonal alienation, mindfulness, and smartphone addiction remains underexplored in the literature. Therefore, this study investigates the relationships among university students’ personality traits, interpersonal alienation, mindfulness, and smartphone addiction, with a focus on mindfulness’s mediating and moderating roles in these relationships. The main objectives are: (1) to examine the correlations between personality traits, interpersonal alienation, mindfulness, and smartphone addiction among students; (2) to assess gender differences in these variables; (3) to test whether mindfulness mediates the relationship between interpersonal alienation and smartphone addiction; and (4) to explore whether mindfulness moderates this relationship.

## Literature review & research hypothesis development

### The essence of personality traits

Personality traits influence a broad spectrum of behaviors [29–32]. The effort to systematically categorize these traits has led to significant frameworks, including the sixteen personality factor questionnaire (16PF) proposed by Cattell and Mead [33], the Eysenck Personality Questionnaire (EPQ) developed by Eysenck and Eysenck [34], and the Big Five personality model introduced by Goldberg [35], delineating Extraversion, Agreeableness, Conscientiousness, Neuroticism, and Openness. These traits are characterized by distinct attributes: extraverts are noted for their enthusiasm in social interactions; agreeable persons are perceived as cooperative and empathic; conscientious individuals are recognized for their organizational skills and responsibility; neurotic individuals tend to experience significant emotional variability; and open individuals are distinguished by their creativity and pursuit of new experiences. The Big Five model has been extensively validated and applied across various studies [36–38], underscoring its broad applicability in personality research.

Recent research has further emphasized the Big Five model as one of the most widely studied and applied frameworks internationally, with multiple studies replicated across countries and cultures. Factor analytic studies using different versions and adaptations of personality measures have consistently supported the five-dimensional structure, and cross-cultural research demonstrates its universality. Even in Latin American contexts, where fewer studies had previously been conducted, recent findings confirm that the Big Five structure is stable and psychometrically reliable across diverse samples, while also preserving cultural uniqueness in individual differences [39]. The significance of personality traits extends across various domains, including entrepreneurship, where recent studies show that both personality traits and entrepreneurship education contribute substantially to entrepreneurial intention, as conceptualized in the theory of planned behaviour [40]. In this context, personality traits serve not only as predictors of individual behavior but also as crucial elements that interact with other psychological attributes.

Moreover, the connection between personality traits and other dispositions, such as mindfulness, has gained increasing attention. Network analysis has provided new perspectives on how personality traits relate to mindfulness and how these interactions can yield further insights into human behavior. Studies have shown that associations between the Big Five factors and facets of mindfulness are consistent across countries, supporting the robustness of these relationships and offering valuable directions for future research and intervention. Although personality research traditionally focuses on similarities and differences among individuals, understanding the processes underlying these traits, including their interactions with mindfulness traits, remains a growing area of interest [41].

### The relationship between personality traits and interpersonal alienation

Kiran and Thiruchelvi [42] identified a significant correlation between the Big Five personality traits and social exclusion, underscoring how personality traits influence perceptions of social exclusion and interpersonal alienation. Lyubyakin [43] contends that loneliness and interpersonal alienation arise from maladaptation and dysfunctions within personality structures, emphasizing the significance of social roles, self-concept, and personality orientation in social adaptation. Teppers et al. [44] found that low agreeableness and high conscientiousness among personality traits predict loneliness. In contrast, a deficiency in extraversion is linked to discomfort with solitude, illustrating the varied effects of personality on feelings of alienation. Gurtman [45] demonstrated that extraversion, agreeableness, and neuroticism notably affect interpersonal relationships. For instance, individuals with low extraversion may experience heightened loneliness due to inadequate social skills [44]; those with elevated neuroticism, susceptible to emotional fluctuations, are more likely to experience loneliness and strained interpersonal functioning [43, 45]; conversely, persons with high agreeableness, owing to their pro-social characteristics, maintain more stable interpersonal relationships and face less alienation [44, 45].

Recent large-scale evidence further emphasizes the importance of neuroticism and loneliness not only in predicting interpersonal alienation but also in influencing health risks and public health outcomes. For example, neuroticism and loneliness are closely associated with premature mortality and intentional self-harm, particularly among younger adults and those with lower education [46]. The strongest associations between personality and cause of death were found for intentional self-harm, as well as respiratory and digestive system diseases, highlighting the public health relevance of these traits [46].

Moreover, research suggests that the relationship between personality and loneliness is dynamic and bidirectional. Joshanloo [47] demonstrated that loneliness predicts subsequent declines in extraversion, agreeableness, and conscientiousness, whereas neuroticism is a positive predictor of future loneliness; other personality traits function as protective factors. Additionally, negative affect is both associated with neuroticism and predicts future loneliness, indicating an interactive process between personality, mood, and feelings of alienation [47].

More nuanced analyses show that individuals high in neuroticism tend to feel lonelier, especially when alone, and experience greater intrapersonal fluctuations in loneliness [48]. These findings support the differential reactivity hypothesis, which posits that individuals vary in their loneliness due to differences in reactivity to social situations, consistent with the conceptualization of neuroticism as a tendency towards heightened sensitivity to social stressors [48]. Based on the reviewed literature, this study proposes:

- Hypothesis 1 (H1): A significant correlation exists between personality traits and interpersonal alienation.

### The relationship between personality traits and smartphone addiction

Smartphone addiction, characterized by an overdependence on smartphones that adversely impacts individuals’ daily activities and educational pursuits, escalates the risk of accidents [49], diminishes life satisfaction [8], degrades sleep quality [50, 51], and undermines both learning efficiency and focus in classroom settings [9, 51, 52]. Marengo et al. [53] identified personality traits as crucial predictors of smartphone addiction risk. Studies indicate that individuals exhibiting elevated levels of neuroticism [54], narcissism [55], loneliness [56], and a propensity for escapism and perceived stress [57] are at heightened risk for developing smartphone addiction. Conversely, agreeableness is inversely correlated with addiction [58, 59], while both neuroticism [53, 60, 61] and extraversion [60, 62, 63] demonstrate a positive correlation.

Recent research further elaborates on the complex associations between personality traits and smartphone addiction. Sun et al. [64] found that neuroticism, loneliness, attachment anxiety, and nomophobia are positively correlated and that neuroticism directly predicts anxiety related to smartphone separation. In this framework, attachment anxiety and loneliness sequentially mediate the relationship between neuroticism and nomophobia, supporting the chain mediation model also implied in the present study. Fu et al. [65] indicated that neuroticism, conscientiousness, openness, and agreeableness each have direct effects on depression, with loneliness and problematic Internet use partially mediating the relationship between neuroticism, conscientiousness, agreeableness, and depression, and fully mediating the relationship between extraversion and depression. These findings highlight the multiple intermediary roles that alienation and loneliness may play in the context of digital addiction.

Lian [66] further demonstrated that alienation mediates the association between interpersonal relationships and smartphone addiction, while conscientiousness directly impacts smartphone addiction regardless of alienation. Moreover, alienation also moderates the link between conscientiousness and smartphone addiction, providing support for the interaction effects hypothesized in this study. Supporting the predictive role of personality, Erzincanli & Geçikli [67] reported a significant negative association between certain personality traits and digital or smartphone addiction, indicating that higher levels of positive personality traits may serve as protective factors. Bhayangkara et al. [68] found that conscientiousness, extraversion, and neuroticism were significantly associated with smartphone addiction, whereas openness and agreeableness were not in their student sample.

Additionally, machine learning models, such as the random forests used by Osorio et al. [69], validate the relationship between adolescents’ personality traits, particularly neuroticism and conscientiousness, and smartphone addiction risk. Yan et al. [70] reported that individuals with higher neuroticism exhibit lower self-control, increasing the likelihood of smartphone addiction. Based on the reviewed literature, this study proposes:

- Hypothesis 2 (H2): A significant correlation exists between personality traits and smartphone addiction.

### The relationship between personality traits and mindfulness

Mindfulness, defined as a psychological practice focused on present-moment experiences without judgment and characterized by openness and acceptance [71], has been shown to confer numerous benefits, including enhanced attention [72], improved sleep quality [73, 74], and effective pain management [75, 76]. The literature suggests a discernible relationship between the Big Five personality traits and mindfulness [77–79], with a notably negative correlation with neuroticism. Individuals exhibiting high levels of neuroticism frequently encounter stress and adverse emotions [80]. In contrast, conscientiousness is positively correlated with mindfulness, likely because individuals with high conscientiousness who pursue efficiency find mindfulness beneficial in enhancing focus [77]. However, the associations between agreeableness, openness, extraversion, and mindfulness are less conclusively established [81, 82].

Recent research further clarifies the link between personality traits and mindfulness. Empirical evidence supports a positive predictive relationship between certain personality traits and mindfulness levels [67]. Specifically, personality structure is considered an important factor in fostering mindfulness among university students, and both personality traits and mindfulness are key determinants in students’ use of digital tools [67]. This underscores the multi-factorial model linking personality and mindfulness to behavioral outcomes.

The association between neuroticism and mindfulness has been extensively studied. Neuroticism, as a psychological risk factor, is negatively associated with mindfulness, a relationship that is consistent across most studies [79, 83]. Higher trait mindfulness often reflects greater emotional stability and self-regulation capacity. Among the Big Five, trait mindfulness shows the strongest negative correlation with neuroticism and the strongest positive correlation with conscientiousness, aligning with self-regulation theory [79]. Trait mindfulness is often conceptualized as relatively stable and characterized by the ability to maintain awareness across contexts [84]. At the same time, recent momentary-level evidence suggests that mindfulness and its emotion-regulatory benefits can fluctuate within persons across situations [85]. Accordingly, the present study treats mindfulness as a dispositional tendency measured by the MAAS while acknowledging that its expression may still vary by context.

Moreover, the relationship between mindfulness and personality may vary by measurement tool. Research using the Five-Factor Mindfulness Questionnaire (FFMQ) finds associations with all five personality dimensions. In contrast, the Mindful Attention Awareness Scale (MAAS) is primarily linked to neuroticism, conscientiousness, and agreeableness [79]. This underscores the importance of accounting for measurement differences when interpreting results and highlights the potential for future facet-level investigations.

In practice, higher trait mindfulness has been shown to reduce automatic and compulsive behaviors, thereby promoting more conscious decision-making about smartphone use. Individuals with higher mindfulness are generally better able to regulate present-moment experiences and respond less reactively to distress [84], demonstrating protective effects on multiple psychological health indicators, including lower internalizing, externalizing, and overall psychopathology scores [86]. Notably, the self-acceptance facet of mindfulness significantly contributes to the association between mindfulness and reduced psychological symptoms.

Overall, these findings reinforce the view that personality traits, particularly neuroticism and conscientiousness, are closely associated with individual differences in mindfulness. In turn, mindfulness may serve as a protective factor for mental health and adaptive behaviors. Based on these findings, the study posits:

- Hypothesis 3 (H3): A significant correlation exists between personality traits and mindfulness.

### The relationship between interpersonal alienation and smartphone addiction

The Compensatory Internet Use (CIU) theory argues that individuals use the Internet as a compensatory psychological mechanism to alleviate negative emotions. Recent empirical studies have established that adolescents’ dependency on smartphones not only precipitates addictive behaviors but also significantly correlates with increased feelings of interpersonal alienation [13]. The interaction between social exclusion and smartphone addiction underscores the role of avoidance behaviors, suggesting that an over-reliance on smartphones for virtual interactions, rather than direct communication, detrimentally affects adolescents’ social relationships and overall life satisfaction [87]. Moreover, reciprocal links among social rejection, social avoidance, and smartphone addiction suggest that socially avoidant responses may further intensify alienation over time [88]. In situations where individuals lack a sense of belonging and social support, they tend to overuse smartphones and social media to satisfy social needs [13].

Building on this foundation, research on the COVID-19 pandemic provides additional perspective. Interpersonal alienation and perceived meaning in life both increased as the epidemic came under control, highlighting the influence of broader social environments on psychological adaptation [89]. Longitudinal evidence indicates that interpersonal alienation not only negatively predicts subsequent meaning in life but also positively predicts later smartphone addiction [89]. Importantly, meaning in life during the intermediate period of the epidemic was found to mediate the relationship between earlier alienation and subsequent smartphone addiction, suggesting a multi-layered mediating process.

The role of loneliness, closely related to interpersonal alienation, has also been examined in depth. For adolescents, loneliness is a common experience, and a wealth of evidence demonstrates a robust link between loneliness and smartphone addiction [90, 91]. Both trait and state-level loneliness have been shown to predict smartphone addiction, with the effect primarily unidirectional [90, 92]. That is, loneliness predicts problematic smartphone use, but problematic smartphone use does not predict loneliness.

Beyond emotional mechanisms, the mediating and moderating roles of alienation have also been noted. Lian [66] found that alienation mediates the relationship between interpersonal factors and smartphone addiction, while also highlighting the importance of protective factors such as conscientiousness. Although much of the research emphasizes the risk pathways, the investigation of potential protective mechanisms, including virtues and positive personality traits, remains limited. Recent work also suggests that the association between social relationships and internet addiction can be understood through both hedonic and eudaimonic well-being pathways, indicating that deteriorated relationships may undermine well-being and in turn heighten compensatory technology use [28].

As smartphone use becomes increasingly prevalent, its impact extends beyond addiction to influence social support and well-being. For instance, the positive effect of sense of coherence on social support weakens with increased smartphone use, reflecting the complex interplay between digital behaviors and psychosocial resources [93]. Related evidence further indicates that sex and perceived social support can alter the association between mental health difficulties and problematic digital behaviors, implying that contextual resources may protect some students more effectively than others [94].

Taken together, these findings support the proposition that interpersonal alienation is a significant predictor of smartphone addiction, operating through multiple psychological mechanisms and contextual factors. Based on these findings, we posit:

- Hypothesis 4 (H4): There is a positive correlation between interpersonal alienation and smartphone addiction.

### The mediating role of mindfulness in interpersonal alienation and smartphone addiction

Recent advances in mindfulness therapy in psychology and the behavioral sciences have demonstrated its efficacy in enhancing interpersonal relationships, reducing interpersonal stress, and increasing social engagement [25, 27, 95]. Individuals with higher levels of mindfulness are often more extroverted and engaged in social contexts [96]. Mindfulness training can strengthen observational skills, empathy, and social efficacy, while reducing social anxiety [97, 98]. Enhanced mindfulness also fosters self-awareness, which supports impulse regulation, including in relation to internet and smartphone use [99].

Empirical research further demonstrates that mindfulness reduces smartphone addiction by improving self-regulation and introspection and by lessening the tendency to use smartphones before sleep [100]. Short-term online mindfulness interventions have been shown to reduce various types of smartphone addiction [101]. Both trait mindfulness and specific mindfulness facets, such as acting with awareness, negatively predict smartphone addiction across multiple addiction types [101]. Groups with problematic smartphone use generally exhibit higher impulsivity and lower mindfulness levels [71]. Mediation analyses indicate that mindfulness, especially the facet of acting with awareness, can reduce problematic smartphone use by lowering attentional impulsivity [71]. Furthermore, increased mindfulness supports the development of a clearer self-concept, which mediates the link between smartphone use and emotional well-being [102].

The relationship between mindfulness and problematic smartphone use is consistently negative [103]. Smartphone involvement partially mediates the effect of smartphone use on mindfulness [104], providing initial empirical support for conceptual models that propose a strong link between mindfulness and highly engaged smartphone use. Targeted interventions that strengthen mindfulness and self-control can mitigate the effects of poor emotion regulation on smartphone addiction. Broader psychological capital also acts as a protective factor in the association between neuroticism, self-control, and smartphone addiction [70, 105]. In addition, meta-analytic evidence suggests that different mindfulness facets are differentially associated with the Big Five, indicating that the linkage between personality and stress-related functioning may vary across mindfulness components [79, 82]. Other work suggests that improvements in trait mindfulness can lead to positive changes in neuroticism, which in turn explain improved mental health outcomes [106]. Multiple studies reveal complex, layered mediation and moderation effects involving mindfulness, self-control, emotion regulation, and psychological well-being. For example, expressive suppression and emotional well-being sequentially mediate the relationship between loneliness and smartphone addiction [91]. Mindfulness-based mechanisms, such as self-compassion, emotion regulation, and cognitive reappraisal, are important to the efficacy of mindfulness interventions [107].

In summary, current evidence is consistent with the view that mindfulness may be associated with a weaker link between interpersonal alienation and smartphone addiction through multiple psychological processes, functioning as both a mediator and a protective factor in this association. Based on the literature review, this study posits:

- Hypothesis 5 (H5): There is a negative correlation between interpersonal alienation and mindfulness.
- Hypothesis 6 (H6): Mindfulness and smartphone addiction correlate negatively.
- Hypothesis 7 (H7): Mindfulness mediates the relationship between interpersonal alienation and smartphone addiction.

### The moderating role of mindfulness in interpersonal alienation and smartphone addiction

Mindfulness, defined as present-moment awareness and acceptance, has been demonstrated to moderate the impact of social isolation and distrust, key elements of Early Maladaptive Schemas (EMSs), on smartphone addiction, thereby attenuating its adverse consequences [108]. Mindfulness interventions can reduce the effect of loneliness on internet addiction [109] and moderate the impact of social exclusion on problematic internet use [110]. Furthermore, mindfulness enhances the quality of interpersonal interactions and alleviates stress, thereby providing a protective effect against addictive behaviors, including smartphone addiction [25, 108, 111].

Recent studies provide further support for mindfulness as a protective moderator. For example, mindfulness can buffer the direct effect of smartphone addiction on academic burnout and the indirect effect of technological conflict on academic burnout [112]. These moderating effects are particularly pronounced among individuals with lower mindfulness levels, highlighting the importance of stratified analyses to understand the impact of mindfulness interventions [112]. Similarly, higher mindfulness is associated with a weaker association between self-regulatory fatigue and smartphone addiction, underscoring its buffering role in risk pathways [113].

Mindfulness also moderates other psychological processes: students with higher mindfulness show a stronger association between academic anxiety and self-regulatory fatigue, while those with lower mindfulness are more susceptible to the negative impact of self-regulatory fatigue on smartphone addiction [113]. This dual effect demonstrates that while mindfulness can enhance certain psychological responses, it also protects against maladaptive outcomes.

Beyond academic settings, mindfulness moderates the link between loneliness and depression or addiction, with more pronounced effects among those with lower mindfulness levels [114]. Furthermore, mindfulness moderates the direct relationship between alexithymia and problematic smartphone use, and this effect is stronger in individuals with higher mindfulness [115]. Evidence from crisis contexts, such as the COVID-19 pandemic, also shows that mindfulness moderates the indirect link between anxiety, loneliness, and negative work performance, suggesting its relevance in both clinical and occupational settings [116]. Notably, the moderating effect of mindfulness can be complex and context-dependent, sometimes incurring psychological costs [116]. In addition, mindfulness serves as a negative moderator in high-risk social environments, reducing the likelihood of information addiction. Mindfulness can also act as a protective buffer in the relationship between psychological capital and smartphone addiction [105].

In summary, current research consistently highlights mindfulness as an important moderating factor that can buffer the negative effects of interpersonal alienation, loneliness, and related stressors on smartphone addiction and related outcomes. These effects may differ across contexts and individual levels of mindfulness, suggesting the value of tailored mindfulness interventions. Based on these findings, we posit:

- Hypothesis 8 (H8): Mindfulness moderates the relationship between interpersonal alienation and smartphone addiction.

### The current study

**Fig. 1:**
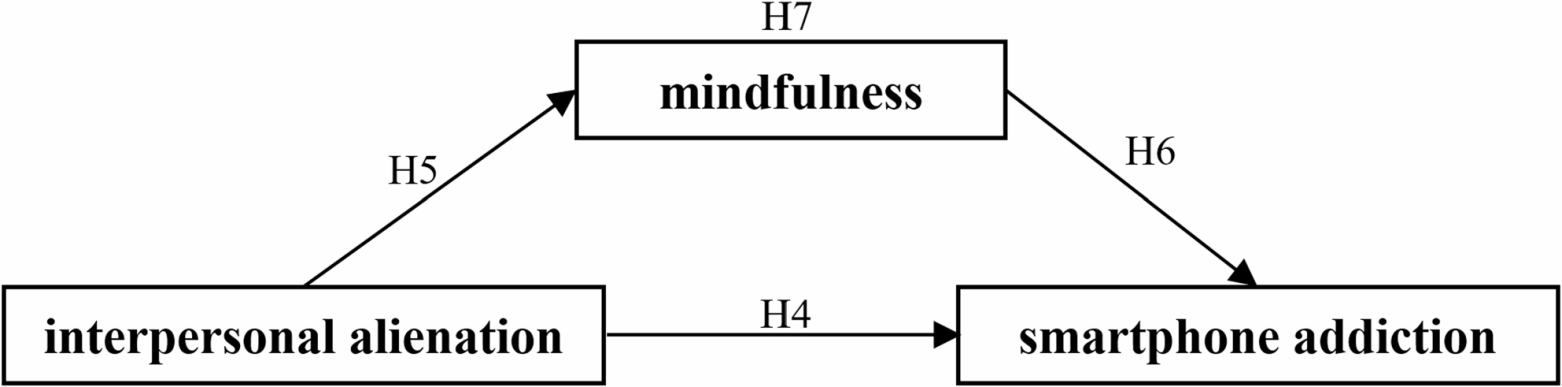
Research model of the mediating effect of mindfulness between interpersonal alienation and smartphone addiction

**Fig. 2:**
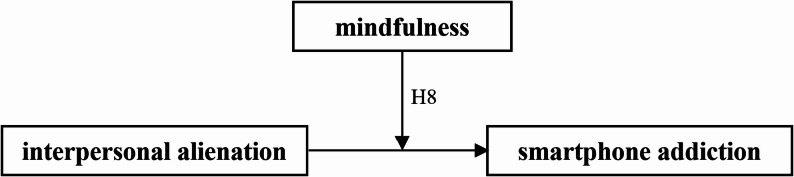
Research model of the moderating effect of mindfulness between interpersonal alienation and smartphone addiction

Building on the preceding literature review and hypothesis development, this investigation focuses on university students in Taiwan to assess a proposed model integrating the Big Five personality traits, interpersonal alienation, mindfulness, and smartphone addiction. In this model, the Big Five traits are treated as dispositional background correlates that help contextualize variation in alienation, mindfulness, and smartphone addiction rather than as deterministic causes. The study examines the relationships among university students’ personality traits, interpersonal alienation, mindfulness, and smartphone addiction. Furthermore, it analyzes variances in personality traits, interpersonal alienation, mindfulness, and smartphone addiction across demographic segments of the university student population. As illustrated in Fig. 1, the research examines the mediating role of mindfulness in the relationship between interpersonal alienation and smartphone addiction. Fig. 2 probes into the moderating effect of mindfulness on the relationship between interpersonal alienation and smartphone addiction.

This study is informed by evidence indicating that these psychological and behavioral variables may interact unidirectionally or bidirectionally within individuals, supporting the proposed model’s examination of both direct and indirect pathways [47]. For example, longitudinal data suggest that interpersonal alienation can negatively predict subsequent meaning in life, which may then mediate the association between alienation and later smartphone addiction, especially during significant contextual shifts such as the COVID-19 pandemic [89]. These findings highlight the value of examining not only direct associations but also potential mediating and moderating processes within the model.

Furthermore, previous research points to the mediating role of loneliness and problematic internet use (PIU) in the relationships between personality traits and negative mental health outcomes, such as depression [65]. Such evidence highlights the value of exploring multi-layered mediation and moderation processes in understanding digital addiction and its psychological correlates. Additional studies reveal that metacognitions about social media use can also mediate the link between negative affect and social media addiction, suggesting the presence of complex, multi-pathway effects within the broader model [117].

Overall, the present research seeks to advance understanding of how personality traits, interpersonal alienation, and mindfulness jointly contribute to smartphone addiction among university students, and to clarify the mediating and moderating functions of mindfulness. The model is designed to capture both static and dynamic aspects of these relationships, reflecting the evolving interplay among personality, cognition, and digital behavior in young adults.

## Method

### Participants and procedures

A web-based survey was conducted among Taiwanese university students in 2022. Sample size estimation was conducted using G*Power 3.1.9.7 [118] with F tests for linear multiple regression using a fixed model with R² deviation from zero. Assuming up to 8 predictors in the most parameterized equation, with an effect size of f² = 0.10 [119], alpha = 0.05, and power = 0.80, the minimum required sample size was 158 participants. The survey was administered from May 10 to 24, 2022, using convenience sampling and was distributed via social media and educational platforms by ten university professors [120].

Convenience sampling is frequently used in educational and behavioral research when time and access constraints make probability-based sampling impractical [121–124]. Although it limits population-level generalizability, it can still provide useful empirical insights when procedures are transparently reported and statistical analyses are conducted rigorously [123, 124].

All procedures complied with the ethical principles of the Declaration of Helsinki issued in 1975 and revised in 2013, and with applicable ethical standards for observational research. Prior to participation, all prospective respondents received a comprehensive explanation of the study objectives and procedures, data management practices, confidentiality safeguards, response anonymity, potential risks, and the right to decline participation or withdraw at any time without penalty. Electronic informed consent was obtained from all participants before completion of the questionnaire. All collected data were deidentified and analyzed solely for research purposes, and any potentially identifiable information was treated as strictly confidential to protect participant rights and welfare throughout the study.

Complete responses were required to reduce data loss, and all entries were thoroughly checked for accuracy. The final dataset comprised 586 valid responses from 67 universities in Taiwan, with 63.7% female (373) and 36.3% male (213) participants, spanning all academic years and both STEM (36.9%, 216) and non-STEM (63.1%, 370) fields.

### Measures

This study employed scales widely documented in academic journals and frequently cited for measurement objectives. The Mindful Attention Awareness Scale and the Smartphone Addiction Scale–Short Version are English-language questionnaires. Five academic experts translated, reviewed, and refined the scales to ensure robust content validity. The back-translation method was utilized to enhance translation accuracy, a common practice in cross-linguistic research to ensure high fidelity between the translated and original text [125].

#### Chinese shortened version of the Big Five Inventory (BFI-15)

The Chinese Shortened Version of the Big Five Inventory (BFI-15), developed by Li and Chung [126], was used to evaluate university students’ Big Five personality traits. Comprising 15 items- three per trait, responses were captured on a Likert scale from 1 (“strongly disagree”) to 5 (“strongly agree”), with higher scores indicating stronger trait presence. The original instrument’s Cronbach’s alpha ranged from 0.67 to 0.81. Confirmatory factor analysis on this study’s sample showed item factor loadings all above 0.7, with fit indices of CFI = 0.951, GFI = 0.938, NFI = 0.956, RMR = 0.060, and RMSEA = 0.071. Composite reliability scores were extraversion (0.924), agreeableness (0.906), conscientiousness (0.890), neuroticism (0.882), and openness (0.887), with corresponding Cronbach’s alphas of 0.874, 0.845, 0.810, 0.799, and 0.801. These findings confirm the strong reliability and validity of the BFI-15 for assessing personality traits among university students.

#### Interpersonal Alienation Scale (IAS)

Based on the work of Yang et al. [127], a three-item version of the Interpersonal Alienation Scale (IAS) was used to measure university students’ perceived interpersonal alienation. This three-item, single-factor scale uses a 7-point Likert response format ranging from 1 (strongly disagree) to 7 (strongly agree), with higher scores indicating greater perceived alienation. The original instrument reported satisfactory internal consistency (Cronbach’s alpha = 0.870). In the current sample, all standardized factor loadings exceeded 0.700. Because a three-item single-factor CFA is just-identified (df = 0), global fit indices such as CFI, GFI, NFI, and RMSEA are not substantively informative and were therefore not emphasized in the evaluation of this scale. Accordingly, we focused on item loadings and reliability evidence. Composite reliability was 0.909, and Cronbach’s alpha was 0.890, supporting the scale’s adequate internal consistency and construct reliability for assessing university students’ interpersonal alienation.

#### Mindful Attention Awareness Scale (MAAS)

The Mindful Attention Awareness Scale (MAAS), developed by Brown and Ryan [84], quantifies mindfulness levels among university students. Comprising 15 items, this scale employs a 6-point Likert response format ranging from “almost always = 1” to “almost never = 6.” This scoring method uses reverse-scored items, so higher total scores indicate a higher level of mindfulness. Subsequent confirmatory factor analysis in this research indicated that all item factor loadings were above 0.700, yielding a CFI of 0.948, GFI of 0.952, NFI of 0.937, RMR of 0.070, and RMSEA of 0.083. The construct reliability reached 0.908, and Cronbach’s alpha was 0.890, evidencing the MAAS’s robust reliability and validity in measuring mindfulness among university students.

#### Smartphone Addiction Scale-Short Version (SAS-SV)

Developed by Kwon et al. [128], the Smartphone Addiction Scale-Short Version (SAS-SV) assesses smartphone addiction among university students. It includes ten items, rated on a Likert scale from “strongly disagree = 1” to “strongly agree = 6,” where higher scores indicate more severe addiction. The original scale’s Cronbach’s alpha was 0.966. Confirmatory factor analysis for this study’s sample showed all items with factor loadings above 0.700, with CFI, GFI, NFI, and RMR at 0.997, 0.996, 0.996, and 0.033, respectively, and RMSEA at 0.076. Composite reliability was 0.921, and Cronbach’s alpha was 0.905, indicating strong reliability and validity for the SAS-SV.

### Data analysis

In this study, AMOS version 25 was used to conduct confirmatory factor analyses of the scales. SPSS version 24 was used to analyze demographic variables and examine differences and associations among personality traits, interpersonal alienation, mindfulness, and smartphone addiction. Pearson correlation analysis was first conducted to provide preliminary bivariate evidence for hypotheses H1 through H6. To further examine the proposed indirect mechanism, mediation effects were tested using Hayes’ PROCESS Model 4, which was used primarily to evaluate H7 while also providing regression-based evidence relevant to H4, H5, and H6. Hayes’ PROCESS Model 1 was then used to test the moderating effect of mindfulness on the relationship between interpersonal alienation and smartphone addiction, thereby examining H8. The mediation and moderation models were estimated separately because mindfulness was conceptualized as serving two theoretically distinct functions in this study: as an explanatory pathway linking interpersonal alienation to smartphone addiction and as a boundary condition shaping the strength of that association.

## Results

### Test the extent of the Common Method Variance (CMV)

To assess the presence of common method variance (CMV), Harman’s single-factor test was applied to an exploratory factor analysis without rotation. The outcome revealed that the leading factor accounted for 37.058% of the overall variance, which did not exceed 50%, indicating that common method variance was unlikely to be a concern [129, 130].

### Correlations among personality traits, interpersonal alienation, mindfulness, and smartphone addiction

Table 1 shows that interpersonal alienation was moderately and positively correlated with smartphone addiction (*r* = 0.386, *p* < 0.001), supporting H4. Mindfulness was moderately and negatively correlated with both interpersonal alienation (*r* = -0.531, *p* < 0.001) and smartphone addiction (*r* = -0.519, *p* < 0.001), thereby supporting H5 and H6. Regarding personality traits, extraversion (*r* = -0.351, *p* < 0.001), agreeableness (*r* = -0.138, *p* < 0.001), and conscientiousness (*r* = -0.210, *p* < 0.001) were negatively correlated with interpersonal alienation, whereas neuroticism was positively correlated with interpersonal alienation (*r* = 0.550, *p* < 0.001); openness to experience was not significantly correlated with interpersonal alienation. For smartphone addiction, neuroticism showed a moderate positive correlation (*r* = 0.410, *p* < 0.001), conscientiousness showed a weak negative correlation (*r* = -0.099, *p* < 0.05), whereas extraversion, agreeableness, and openness to experience were not significantly correlated with smartphone addiction. In relation to mindfulness, extraversion (*r* = 0.236, *p* < 0.001), agreeableness (*r* = 0.139, *p* < 0.001), and conscientiousness (*r* = 0.281, *p* < 0.001) were positively correlated with mindfulness, while neuroticism was negatively correlated with mindfulness (*r* = -0.485, *p* < 0.001); openness to experience was not significantly correlated with mindfulness. Overall, these bivariate findings provided preliminary support for H4, H5, and H6, and partial support for H1, H2, and H3.

**Table 1:** Correlations among personality traits, interpersonal alienation, mindfulness, and smartphone addiction (*N* = 586)

	M	SD	IA	SA	MI	EX	AG	CO	NE		OP
1. IA	3.029	1.556	1								
2. SA	3.395	1.198	0.386***	1							
3. MI	4.059	0.844	-0.531***	-0.519***	1						
4. EX	3.430	1.081	-0.351***	-0.024	0.236***	1					
5. AG	4.025	0.834	-0.138***	0.021	0.139***	0.545***	1				
6. CO	3.981	0.811	-0.210***	-0.099*	0.281***	0.524***	0.492***	1			
7. NE	3.203	1.106	0.550***	0.410***	-0.485***	-0.310***	-0.043	-0.157***	1		
8. OP	3.514	0.968	-0.028	0.016	0.051	0.257***	0.359***	0.407***	-0.008		1

*IA* Interpersonal alienation, *SA* Smartphone addiction, *MI* Mindfulness, *EX* Extraversion, *AG* Agreeableness, *CO* Conscientiousness, *NE* Neuroticism, *OP* Openness

*p < 0.05, **p < 0.01, ***p < 0.001.

### Gender differences in interpersonal alienation, mindfulness, and smartphone addiction

Table 2 shows that male participants’ scores in interpersonal alienation (M = 3.247, SD = 1.599) were higher than those of females (M = 2.903, SD = 1.515), with t(584) = 2.588, *p* < 0.01, indicating a significant difference. However, no significant differences were found between males and females in smartphone addiction (males: M = 3.442, SD = 1.189; females: M = 3.368, SD = 1.203; t(584) = 0.715, *p* > 0.05) or mindfulness (males: M = 3.999, SD = 0.903; females: M = 4.093, SD = 0.860; t(584) = -1.299, *p* > 0.05).

**Table 2:** Analysis of gender differences in interpersonal alienation, mindfulness, and smartphone addiction (*N* = 586)

Gender	N	IA			SA			MI		
		M	SD	*t*	M	SD	*t*	M	SD	*t*
Male	213	3.247	1.599	2.588**	3.442	1.189	0.715	3.999	0.903	-1.299
Female	373	2.903	1.515		3.368	1.203		4.093	0.860	

*IA* Interpersonal alienation, *SA* Smartphone addiction, *MI* Mindfulness

**p < 0.01. df = 584 for all independent-samples t tests.

### Mindfulness as a mediator between interpersonal alienation and smartphone addiction

**Fig. 3:**
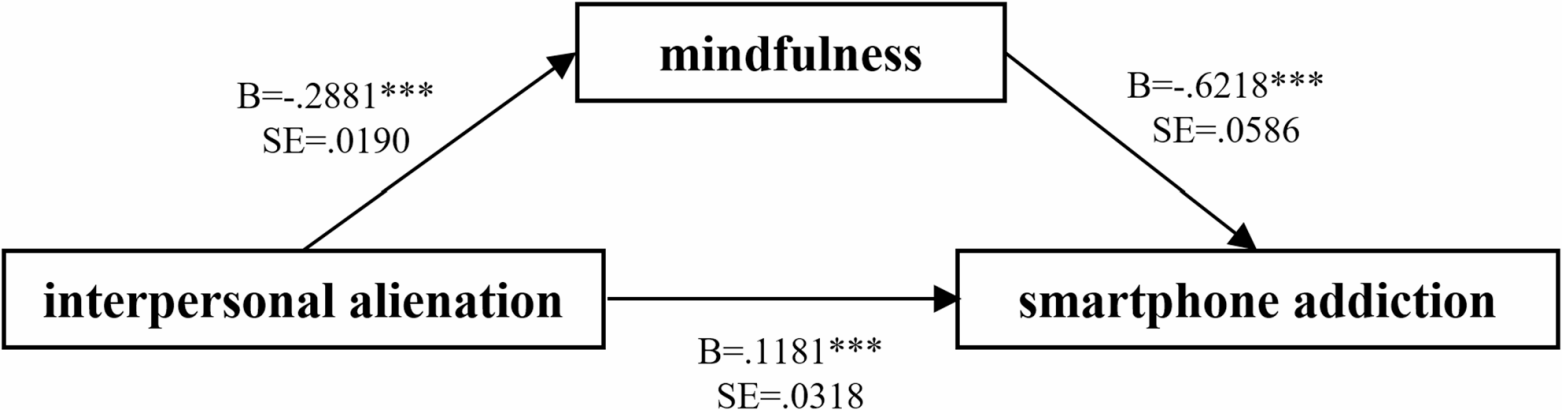
The mediating role of mindfulness in the relationship between interpersonal alienation and smartphone addiction. *** *p* < 0.001

Table 3; Fig. 3 summarize the mediation model examining whether mindfulness statistically mediates the association between interpersonal alienation (IA) and smartphone addiction (SA). IA showed a significant positive total effect on SA (B = 0.297, SE = 0.029, *t* = 10.113, 95% CI [0.240, 0.355]), indicating that higher interpersonal alienation was associated with greater smartphone addiction and supporting H4. After mindfulness was entered as a mediator, the direct effect of IA on SA remained statistically significant but was attenuated (B = 0.118, SE = 0.032, *t* = 3.714, 95% CI [0.056, 0.181]), suggesting that mindfulness explains part, but not all, of the IA–SA association.

The pattern of path coefficients was also consistent with the proposed mediation model. IA was negatively associated with mindfulness (B = -0.288, SE = 0.019, *p* < 0.001), supporting H5, and mindfulness was negatively associated with SA (B = -0.622, SE = 0.059, *p* < 0.001), supporting H6. Importantly, the indirect effect of IA on SA via mindfulness was significant (B = 0.179, Boot SE = 0.021, 95% bootstrap CI [0.138, 0.221]), with the bootstrap confidence interval excluding zero, thereby supporting H7. Collectively, these results indicate a partial mediation: interpersonal alienation is associated with lower mindfulness, which in turn is linked to higher smartphone addiction, while a residual direct effect of interpersonal alienation on smartphone addiction persists even after accounting for mindfulness.

**Table 3:** The mediating role of mindfulness in the relationship between interpersonal alienation and smartphone addiction

	B	SE	t	LLCI	ULCI
Total effect of IA on SA	0.297	0.029	10.113	0.24	0.355
Direct effect of IA on SA	0.118	0.032	3.714	0.056	0.181
Indirect effect of IA on SA	B	Boot SE		Boot LLCI	Boot ULCI
Indirect effect of IA on SA via mindfulness	0.179	0.021		0.138	0.221

Bootstrap confidence intervals are reported for the indirect effect

*IA* Interpersonal alienation, *SA* Smartphone addiction

### The moderating effect of mindfulness on the relationship between interpersonal alienation and smartphone addiction

Table 4 presents the moderation model predicting smartphone addiction from interpersonal alienation, mindfulness, and their interaction. To facilitate interpretation, interpersonal alienation and mindfulness were mean-centered, and the interaction term was computed as the product of the centered variables. Accordingly, the main effects reported in Table 4 should be interpreted as conditional effects evaluated when the other centered predictor is at its mean, rather than as coefficients directly comparable to those from the mediation model in Table 3. Results indicated that, holding the other predictors constant, interpersonal alienation was a significant predictor of smartphone addiction (B = 0.287, t = 2.395, *p* < 0.05, 95% CI [0.052, 0.522]), indicating a positive association at mean mindfulness. Mindfulness was also negatively associated with smartphone addiction (B = -0.934, t = -8.787, *p* < 0.001, 95% CI [-1.142, -0.725]), indicating that higher mindfulness was associated with lower smartphone addiction when interpersonal alienation was at its mean. Importantly, the interaction term between interpersonal alienation and mindfulness was significant and positive (B = 0.102, t = 3.504, *p* < 0.001, 95% CI [0.045, 0.160]), and its confidence interval did not include zero, indicating a reliable moderating effect. This pattern suggests that mindfulness moderated the association between interpersonal alienation and smartphone addiction, such that the positive association between interpersonal alienation and smartphone addiction strengthened as mindfulness increased, shifting the slope toward a more positive direction at higher levels of mindfulness. In parallel, the protective association of mindfulness with smartphone addiction became weaker as interpersonal alienation increased, consistent with a reverse-buffering pattern in which the relative protective advantage of mindfulness is compressed under stronger relational strain rather than reversed into a harmful effect.

**Table 4:** The moderation effect testing of mindfulness on interpersonal alienation and smartphone addiction

variable	B	t	LLCI	ULCI
Interpersonal alienation	0.287	2.395*	0.052	0.522
Mindfulness	-0.934	-8.787***	-1.142	-0.725
Interpersonal alienation × mindfulness	0.102	3.504***	0.045	0.160

*p < 0.05, **p < 0.01, ***p < 0.001

**Fig. 4:**
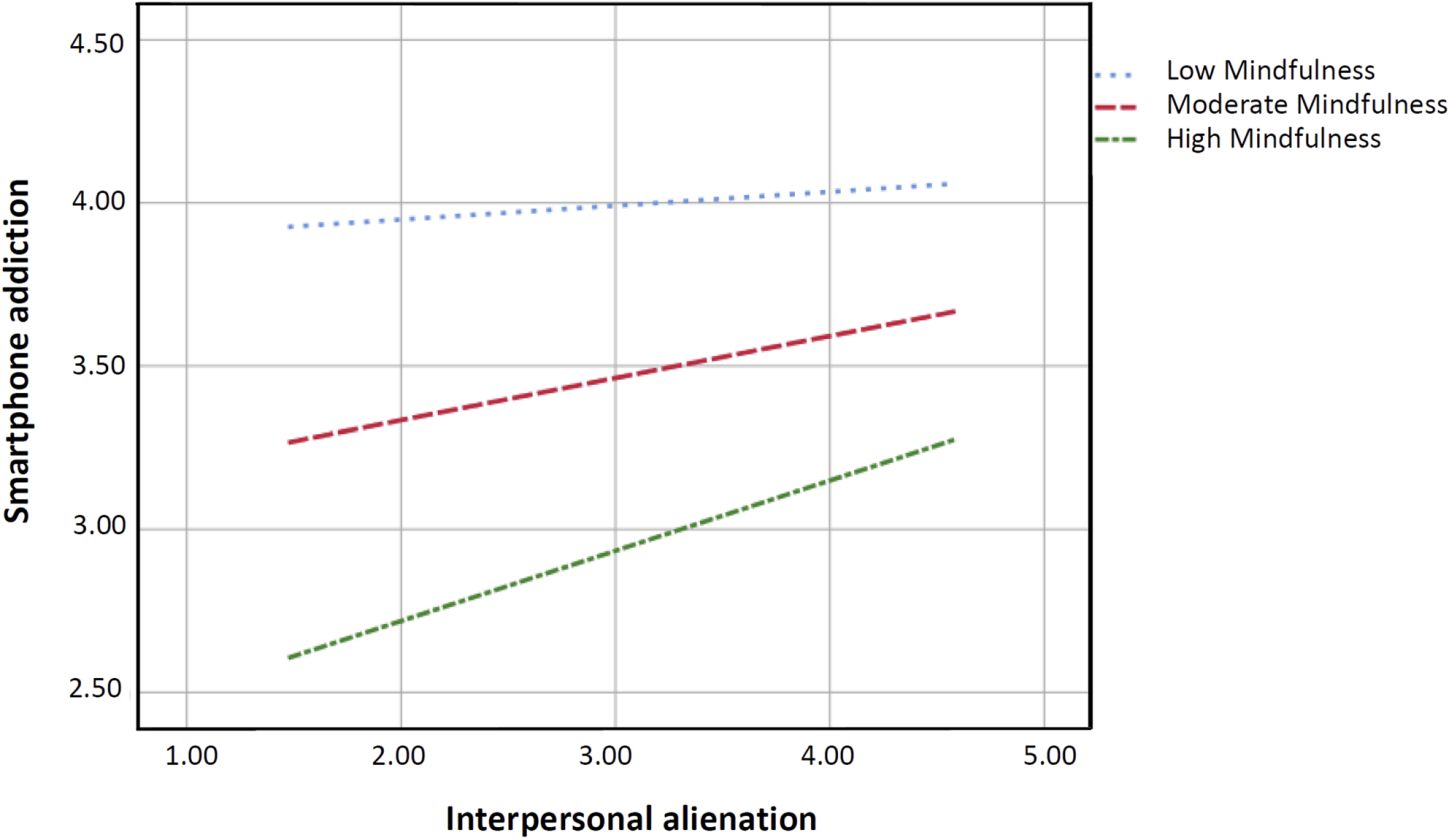
The effect of interpersonal alienation on smartphone addiction moderated by mindfulness

Figure 4 illustrates this conditional pattern by plotting predicted smartphone addiction across interpersonal alienation at low, moderate, and high levels of mindfulness. Two features are evident. First, higher mindfulness is associated with lower predicted smartphone addiction across the range of interpersonal alienation, with the high-mindfulness line lowest, the moderate-mindfulness line intermediate, and the low-mindfulness line highest. This separation is most pronounced when interpersonal alienation is low. Second, the association between interpersonal alienation and smartphone addiction varies as a function of mindfulness, as indicated by the nonparallel simple slopes. Interpersonal alienation acts as a compressive force that narrows the protective space mindfulness affords. In these high-alienation scenarios, the sharp rise in addiction risk suggests that the ‘mindfulness buffer’ is being squeezed, not that the trait itself has lost its value. Consequently, the gap between the three lines narrows as interpersonal alienation increases, suggesting that the protective association of mindfulness with smartphone addiction weakens at higher levels of interpersonal alienation.

## Discussion

### Unveiling how mindfulness influences the relationship between interpersonal alienation and smartphone addiction

This investigation established a moderate positive correlation between interpersonal alienation and smartphone addiction, supporting the findings of Wang et al. [13]. It also aligns with longitudinal evidence showing that social rejection, social avoidance, and smartphone addiction can reinforce one another over time [88]. Moreover, the study identified a moderate negative relationship between mindfulness and both alienation and addiction, indicating that higher mindfulness was associated with lower alienation and lower addictive tendencies, in concordance with Altinyelken [131]. Mindfulness interventions have been shown to enhance psychosocial well-being and interpersonal relationships, offering a potential avenue for mitigating smartphone addiction [2, 26]. Regarding personality traits, the research found small but significant effects of extraversion and agreeableness on mindfulness, whereas conscientiousness was more strongly associated with mindfulness. This pattern suggests that personality traits were associated with differences in mindfulness, which is consistent with prior work on the Big Five and mindful personality structure [41, 77, 79]. Additionally, the moderate negative correlation between neuroticism and mindfulness suggests that emotional instability may be linked to stronger addictive tendencies.

### The role of gender in shaping the trajectories of interpersonal alienation, mindfulness, and smartphone addiction

The current study found that men exhibited significantly higher levels of interpersonal alienation than women, highlighting gender differences in emotional regulation and social interaction [42, 43]. Additionally, our analysis found no significant gender differences in mindfulness and smartphone addiction, underscoring the pervasive role of smartphones in modern life and suggesting that mindfulness may remain broadly relevant as a supportive approach to psychological well-being [131]. Importantly, our findings resonate with Wu & Chou [1], showing no direct link between gender and smartphone addiction but a significant relationship between addiction and insecure interpersonal attachment, advocating for the cultivation of healthy interpersonal relations to combat excessive smartphone use. Despite gender differences in levels of alienation, the negligible differences in smartphone addiction and mindfulness efficacy underscore the essential role of mindfulness and adjunct psychosocial factors, such as social support and skills, in addressing alienation and reducing smartphone addiction [13].

### Exploring the mediating role of mindfulness between interpersonal alienation and smartphone addiction

This study clarified the link between interpersonal alienation and smartphone addiction by revealing a strong total but weaker direct effect, indicating mediation. Mindfulness emerged as a key mediator: individuals with greater nonjudgmental present-moment awareness reported lower smartphone addiction, consistent with findings that mindfulness is inversely associated with problematic use [11, 131]. Mindfulness likely mitigates maladaptive reliance on smartphones by enhancing emotional and behavioral self-regulation, thereby buffering the negative impact of alienation. Prior research shows that mindfulness training improves social observation and empathy and reduces social anxiety, thereby lowering the risk of addiction [97, 98]. Social pressures also influence this process, as peer pressure predicts both anxiety and smartphone addiction, emphasizing the need to target emotional pathways in interventions [10]. Although prior studies have reported that agreeableness may be negatively associated with problematic or addictive technology use [132, 133], this pattern was not observed in the present data. Together, these findings support mindfulness-based strategies and underscore the importance of addressing personality risks and emotionally charged social contexts. Expanding this framework, Sun et al. [134] identified loneliness as a central mediator between psychological need satisfaction and smartphone addiction, revealing a sequential path where both factors jointly contribute to addiction risk and highlighting the need to address unmet needs and loneliness in prevention efforts.

### Mindfulness as the power of moderating interpersonal alienation and smartphone addiction

The present study indicates that mindfulness functions as a salient psychological resource in relation to smartphone addiction, particularly in interpersonal contexts marked by alienation. Consistent with prior evidence that mindfulness is negatively associated with problematic smartphone use [11], higher mindfulness in our data was associated with lower addictive tendencies, which may reflect greater attentional control, stronger self-regulation, and less rumination that otherwise heighten vulnerability to maladaptive technology use [100, 107]. Interpersonal alienation has been identified as a key risk factor for smartphone addiction [13], and socially aversive experiences can motivate compensatory coping behaviors, including excessive online engagement, when individuals struggle to regulate negative affect effectively [135]. Importantly, the moderation pattern observed here is better interpreted as a reverse-buffering pattern than as evidence that mindfulness becomes harmful under high alienation. Across the observed range of interpersonal alienation, higher mindfulness remained associated with lower predicted smartphone addiction, but the distance between the low-, moderate-, and high-mindfulness lines narrowed as alienation increased. In other words, mindfulness retained a protective association, yet its relative advantage was compressed under stronger relational strain. This interpretation is compatible with recent work showing that an apparent protective resource may weaken, or even intensify adverse outcomes, under sufficiently severe stress exposure [136]. It is also consistent with newer resilience research suggesting that contextual resources do not uniformly buffer adversity and may sometimes amplify strain, depending on the type and intensity of adverse experiences [137]. In the present case, the interaction appears closer to a vulnerability process than a deterioration process: heightened alienation may tax self-regulatory resources and strengthen compensatory smartphone use, thereby reducing the extent to which mindfulness can offset addiction pathways, rather than turning mindfulness itself into a risk factor. This reading is also broadly compatible with evidence that social support and other contextual resources can differentially condition problematic digital behaviors [94]. Taken together, these findings suggest that mindfulness-based approaches may be most effective when combined with efforts to reduce social anxiety, repair relational disconnection, and strengthen supportive peer environments.

## Implications

### Theoretical Implications

This research supports the theory of compensatory internet use [135], indicating that individuals may turn to smartphones to cope with interpersonal alienation, thereby increasing the risk of dependence. Results showed a moderate positive association between interpersonal alienation and smartphone addiction, with mindfulness serving as an important protective correlate. This finding is consistent with prior evidence [138] and supports mindfulness as a relevant strategy for reducing psychological discomfort and smartphone overuse. The moderation result further suggests that mindfulness does not operate as a uniformly strong protective factor across all interpersonal contexts. Rather, the observed interaction is more consistent with a reverse-buffering pattern, whereby the protective association of mindfulness is attenuated as interpersonal alienation increases [136, 137]. The study’s findings further highlight the complex, multi-layered relationships among personality traits, mindfulness, alienation, and smartphone addiction. Specifically, conscientiousness was associated with greater mindfulness, and mindfulness was in turn associated with lower interpersonal alienation and lower smartphone addiction [139]. These findings extend prior work by clarifying how personality-oriented mindfulness training can be linked to problematic smartphone use, a claim echoed by Fu et al. [65], who also emphasize the mediating roles of loneliness and problematic internet use in the relationship between personality traits and well-being. Moreover, the study examines the relationship between smartphone addiction and interpersonal alienation, consistent with Hu and Xiang [90], who demonstrate both the importance and practical significance of interventions targeting these dynamics in adolescents. The results also reinforce Lian’s [66] advocacy for interventions that develop virtues, such as conscientiousness, to reduce alienation and digital addiction.

By confirming the mediating role of mindfulness between interpersonal alienation and smartphone addiction, this research strengthens the theoretical foundation for integrating mindfulness-based interventions (MBIs) into prevention and treatment strategies. This is consistent with recent meta-analyses and experimental studies showing that even brief online mindfulness training can effectively reduce problematic mobile phone use [101]. Additionally, the results provide evidence for gender differences in alienation, with men reporting greater interpersonal alienation than women [1, 46]. In contrast, the absence of significant gender differences in the levels of mindfulness and smartphone addiction underscores the broad relevance of mindfulness interventions for mental health. At the same time, the reverse-buffering pattern indicates that mindfulness may be less effective as a stand-alone resource for students experiencing severe relational strain, which supports combining mindfulness with relational or social-support-based interventions.

This study extends understanding of how mindfulness may be linked to lower smartphone addiction, potentially through stronger self-regulation, lower rumination, and reduced social anxiety [97, 100], as well as greater openness and capacity to let go of unpleasant experiences [140]. It also supports exploration of the cross-domain applications of mindfulness in therapy, education, and workplace contexts [141], with current research trends indicating both theoretical growth and practical scalability. Theoretical advances further underscore the value of multifaceted, personality-tailored interventions [47, 66, 139]. Future research should investigate how mindfulness interventions adapted to specific personality profiles can more effectively lower interpersonal alienation and smartphone addiction, while also examining when situational fluctuations in mindfulness may alter the strength of these associations [85].

### Educational implications

Educational institutions are encouraged to integrate mindfulness training into student support systems, not only to strengthen behavioral awareness and stress management but also to reduce interpersonal alienation and smartphone dependence [109, 142]. Evidence indicates that group-based mindfulness-oriented interventions and emotion-regulation-focused supports may further assist adolescents and university students in managing the emotional and behavioral roots of smartphone addiction [2, 107]. Related work also suggests that anxiety-related smartphone use patterns are intertwined with family relational contexts, which should be considered in educational support design [143]. However, the present moderation finding suggests that mindfulness should not be treated as a stand-alone remedy for students with high interpersonal alienation. When relational disconnection is severe, the protective association of mindfulness may weaken, so campus programs should combine mindfulness with strategies that rebuild peer connectedness, counseling access, and supportive social environments [136, 137].

The research underscores the need for multifaceted intervention strategies that promote positive personality traits, such as conscientiousness and self control, address negative affect, and develop social skills [47, 65, 66]. Programs should aim to alleviate loneliness and smartphone overuse as part of comprehensive efforts to improve psychological well-being among adolescents and university students [92, 115]. Tailored interventions should consider individual differences in personality, gender, developmental stage, and social support, because contextual resources do not appear to function uniformly across students and may alter the strength of problematic digital behavior pathways [94].

In practice, brief online mindfulness programs have been shown to reduce problematic mobile phone use among young people [101], and online socio-emotional or mindfulness-based training can also help reduce loneliness [144]. Mindfulness fosters greater attention control and disrupts automatic behavioral patterns [101], while emotional regulation skills can be reinforced through targeted health education curricula. Given broader evidence linking digital-screen use patterns to adolescent mental well-being, collaboration among educators, parents, and health professionals remains important for promoting healthier face-to-face interaction habits and moderate smartphone use [145].

In addition, educational technology and digital health applications that promote mindfulness should account for personality factors such as conscientiousness and neuroticism in their design to better tailor interventions to the needs of diverse students [139]. Attention to physiological and behavioral data [146] can support early detection and individualized support, further strengthening the preventive framework within higher education.

Overall, these findings lay a practical and theoretical foundation for developing scalable, targeted, and evidence-based interventions that improve students’ psychosocial adjustment, reduce alienation, and prevent digital addiction.

## Limitations and future directions

This study possesses several limitations. Firstly, the use of non-random sampling and self-reported measures may introduce sampling bias and reporting errors. Secondly, the cross-sectional design provides only a single time-point snapshot, limiting the ability to draw causal inferences; future research should employ longitudinal or experimental methods to validate the findings. Thirdly, the diversity and intensity of participants’ smartphone app use were not comprehensively examined, potentially overlooking nuanced behavioral patterns. Additionally, the study’s focus on Taiwanese university students constrains the generalizability of findings to other populations and cultural contexts.

To address these limitations, future research should expand the sample to include participants from different regions, age groups, and educational backgrounds, thereby enhancing generalizability. For instance, recent findings suggest that neuroticism and loneliness are particularly predictive of negative health outcomes among younger adults and those with lower educational attainment [46], underscoring the importance of subgroup analyses and more representative samples. Cross-cultural research, including non-WEIRD populations, is also needed to clarify how personality and social context jointly shape experiences of loneliness and addiction [48, 77].

Moreover, researchers are encouraged to deepen the theoretical conceptualization and measurement of mindfulness, as current definitions and instruments may not fully capture its complexity [78, 106]. Future research should refine the construct of trait mindfulness both theoretically and methodologically and examine facet-level associations with personality traits [77, 78].

In intervention research, the duration and intensity of mindfulness practice warrant attention. Evidence suggests that very brief interventions, such as a single 15-minute session, may be insufficient to produce robust psychological changes. Thus, studies should consider optimal intervention lengths and follow-up assessments to gauge sustained effects. Lastly, while the value of mindfulness has been recognized across disciplines, its application in fields such as management and business remains limited, suggesting opportunities for cross-domain research linking mindfulness, stress, and organizational behavior [141]. Future studies should also examine the integration of mindfulness into personality psychology, particularly with respect to personality meta-traits and their impact on digital behaviors.

Expanding the diversity of research participants, refining theoretical and methodological approaches, and extending cross-disciplinary applications will collectively enhance the relevance and impact of future investigations into the complex links among personality, mindfulness, alienation, and smartphone addiction.

## Conclusions

This study examined the associations among Big Five personality traits, interpersonal alienation, mindfulness, and smartphone addiction in a sample of university students, with a focus on the mediating and moderating roles of mindfulness. Extraversion and agreeableness were positively associated with mindfulness and negatively associated with interpersonal alienation. Conscientiousness was positively associated with mindfulness and negatively associated with interpersonal alienation and smartphone addiction. In contrast, neuroticism was positively associated with interpersonal alienation and smartphone addiction and negatively associated with mindfulness. Mindfulness partially mediated the association between interpersonal alienation and smartphone addiction, suggesting that alienation may increase addiction risk partly through diminished self-regulatory capacity. Mindfulness also moderated this association in a context-dependent manner: although higher mindfulness was generally associated with lower smartphone addiction, its protective effect weakened as interpersonal alienation increased. These findings suggest that universities should simultaneously strengthen students’ self-regulation and relational support. Mindfulness-based programs may help reduce smartphone addiction, but their benefits may be maximized when combined with efforts to reduce interpersonal alienation and enhance social connectedness. Future research should incorporate additional determinants, apply longitudinal or experimental designs, and test generalizability across cultural contexts and educational stages.

## Data Availability

The data that support the findings of this study are available from the corresponding author upon reasonable request.
